# Anatomy and Biomechanics of the Unstable Shoulder

**DOI:** 10.2174/1874325001711010919

**Published:** 2017-08-31

**Authors:** Ricardo Cuéllar, Miguel Angel Ruiz-Ibán, Adrián Cuéllar

**Affiliations:** 1 Deparment of Traumatology and Orthopaedic Surgery of Universitary Donostia Hospital San Sebastián, Spain; 2 Departaments of Traumatology and Orthopaedic Surgery of the Universitary Ramon and Cajal Hospital Madrid, Spain; 3 Deparment of Traumatology and Orthopaedic Surgery of Galdácano-Usánsolo Hospital Galdácano, Spain

**Keywords:** Ligaments, Physiology of stability, Propioception, Physiopathology of the glenohumeral instability, Shoulder anatomy, Therapeutic implications

## Abstract

**Purpose:**

To review the anatomy of the shoulder joint and of the physiology of glenohumeral stability is essential to manage correctly shoulder instability.

**Methods:**

It was reviewed a large number of recently published research studies related to the shoulder instability that received a higher Level of Evidence grade.

**Results:**

It is reviewed the bony anatomy, the anatomy and function of the ligaments that act on this joint, the physiology and physiopathology of glenohumeral instability and the therapeutic implications of the injured structures.

**Conclusion:**

This knowledge allows the surgeon to evaluate the possible causes of instability, to assess which are the structures that must be reconstructed and to decide which surgical technique must be performed.

## 1 INTRODUCTION

The glenohumeral joint of the shoulder has the highest range of motion of the human body. It is also the most inherently unstable, being the joint that most often develops recurrent instability.

Different anatomical studies regarding the glenohumeral instability have tried to establish a relationship between the different patterns of glenohumeral instability and the presence of different pathologic conditions. The objective is to adopt treatment protocols, establish guidelines for surgical treatment of lesions of the different injured static stabilizers and define rehabilitation protocols for the cases in which there is deficit or imbalance of the dynamic stabilizing elements. Glenohumeral stability depends on the combination of various factors that can be grouped in capsulo-ligamentary or static stabilizers and musculotendinous or dynamic stabilizers. After the popular work of Turkel special consideration has been given to the static capsulo-ligamentary elements, as these are considered to be the main stabilizers of the glenohumeral joint. Thus injury or failure of these elements is considered responsible for shoulder instability [1].

Moreover, the proprioception mechanisms of the shoulder joint have been investigated and found to be closely related to the response of the muscle stabilizers (dynamic or active). Various histological studies in anatomical specimens have confirmed the presence of afferent nerve endings (Ruffini corpuscles and Pacini) and their distribution in the shoulder capsulo-ligamentary complex [2].

 The dynamic joint stability is coordinated by the central cortical control exercised over local muscle-tendon units. Starting from the proprioceptive signal originating in these receptors, a coupled and optimal feedback of the musculature scapulohumeral is generated. This muscular reaction keeps the humeral head centered relative to the glenoid [3].

Shoulder instability can be due to injury and/or failure of the static capsulo-ligamentary stabilizing elements, which are supporting the sensitive afferent endings. This alteration of the passive stabilizing elements causes a disturbance or delay in the delivery of the proprioceptive signal.

 This delayed signal is transmitted through the sensitive afferent channels causing a delay and/or error of coordination of the corresponding muscle response [4-6]. The dynamic stabilizing mechanism is thus altered and the loss of joint congruity is facilitated.

 The avulsion or tearing of the labrum in association with the capsule and glenohumeral ligaments in the anterior shoulder, known as the Bankart lesion [7], is a result of the shoulder dislocating anteriorly. Lack of healing of the lesion or its progression to the inferior part of the joint is an important factor in instability recurrence. The more severe and extensive the capsular-labral injury is and the larger the capsular recess grows, the greater the disturbance of the proprioception joint mechanisms and, consequently, the greater the degree of instability.

## 2 ANATOMY

The humeral head and the glenoid fossa form the glenohumeral joint. Unlike the hip joint in which the femoral head sits completely inside the acetabulum, the humeral head sits on a shallow glenoid fossa that does not cover it at all. In the absence of bone coverage, stability depends on soft tissues that function as static or dynamic stabilizers: the capsule, glenohumeral ligaments, the rotator cuff, the long head of the biceps and the labrum.

The usual shape of the glenoid is an inverted comma. The cartilage has a concave form with a less thickness of cartilage in the center of the glenoid, bing this point the place of greatest contact with the humeral head. Most glenoid surfaces have a retroverted orientation of 7º and a cranial inclination greater than 15º [8]. The humeral head has a 30º retroversion and a 130-150º inclination relative to the shaft [9].

### 2.1 Gleno-Humeral Capsule

It expands from the glenoid to the anatomical neck of the humerus. The glenoid insertion may vary in its point of origin. On this basis, we can classify the capsule in 2 types: in type I (80% of shoulders) the capsule originates in the labrum, while in type II (20% of shoulders) the capsule originates more medially in the glenoid neck [10]. In Type I, the capsular synovial recesses are small and the anterior capsule is thick. By contrast in type II the synovial recesses are larger and the capsule is less resistant as a whole.

The glenohumeral capsule is large, during the normal joint movement it is lax and does not limit movement, allowing a wide range of motion. When the shoulder is approaching the limits of the normal range of motion the capsule tightens gradually and exerts a stabilizing role.

 When it is tight, it provides mechanical stability to the shoulder and prevents the head from exceeding the limits of stability. The capsule also plays a key role in maintaining stability as it contains the proprioception afferents that help in keeping the head centered by sending nerve signals to the muscles to contract and avoid dislocation [11].

The capsule, except in the inferior part, is reinforced by the rotator cuff. Furthermore, there are thickened zones in the capsule that constitute the glenohumeral ligaments. Additionally other extraarticular ligaments contribute to the glenohumeral stability: coraco-humeral, coraco-acromial and transverse humeral ligament.

### 2.2 Transverse Humeral Ligament

It is made of capsular fibers that span form the lesser tuberosity to the greater tuberosity. This ligament has no direct stabilizing function, but keeps the long head of the biceps in the bicipital groove.

### 2.3 Coraco-Humeral Ligament

This ligament originates on the lateral side of the base of the coracoid and inserts on the greater tuberosity. Due to its transverse arrangement, it has the function to suspend the humeral head when the arms hang, but when the shoulder is in abduction, this ligament relaxes and ceases to have a role in humeral suspension.

### 2.4 Coraco-Acromial Ligament

This ligament runs from the anterior edge of the acromion to the tip of the coracoid. Its function is to help stabilize the humeral head avoiding anterior and inferior translation.

### 2.5 Gleno-Humeral Ligaments (GHL)

These are thickenings of the anterior and inferior portion of the joint capsule and exert their function based on the direction of its fibers. They do not act in all possible movements of the shoulder but only in certain positions. The Glenohumeral ligaments are three: superior, middle and inferior.

### 2.6 Superior Glenohumeral Ligament

The glenoid origin varies: sometimes it has a common origin with the middle glenohumeral ligament, in other subjects, it arises at the same place that the biceps tendon and in others somewhere in between these two structures. From its insertion, it extends to a small fossa slightly superior to the lesser tuberosity.

 It runs through the rotator interval formed between the subscapularis and supraspinatus. Its thickness is greatly variable: in a few cases it may become almost as thick as the biceps tendon, and can be confused with it. By contrast, in other cases it is only a small capsular thickening that is barely noticeable under arthroscopic view.

Due to its trajectory, it is tight in flexion, extension, adduction and external rotation [12]. When tight it limits the posterior and inferior displacement of the humeral head. However, the anatomical studies suggest that it is the GHL that is less relevant in glenohumeral stability [13].

### 2.7 Middle Glenohumeral Ligament

Its origin is in the anterior superior glenoid rim and it inserts in the anterior face of the humerus between the greater tuberosity and the humeral articular surface. From an intra-articular arthroscopic view, it is identified in front of the subscapularis tendon.

The thickness of this ligament is also very variable: it can be thick and tendon like, but can also be very thin or even absent. Its absence seems to be a risk factor for recurrence in cases of anterior dislocation [14].

It becomes tight in external rotation and abduction up to 45º; past this point the inferior glenohumeral ligament is more relevant [15]. Its main function is to prevent anterior translation of the humeral head.

### 2.8 Inferior Glenohumeral Ligament (IGHL)

**Fig. (1):**
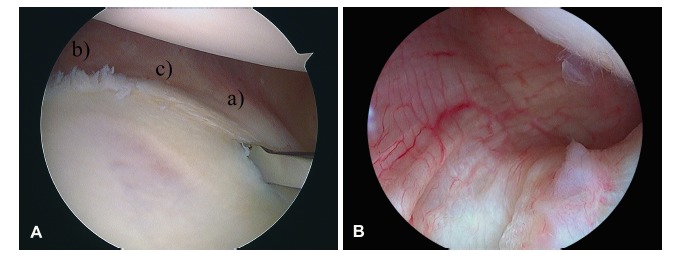
Arthroscopic view of a left shoulder through a posterior portal. **A)** The anatomy of the inferior recess of the glenohumeral joint can be seen. The inferior glenohumeral ligament can be identified in all its components: a) anterior band; b) posterior band; c) axillary pouch. Right shoulder; supero-lateral portal view with a 30-degree scope. **B)** A similar, more posterior view in which the posterior band of the inferior glenohumeral ligament is better appreciated.

It is the thickest and most important shoulder ligament and the main static stabilizer during shoulder abduction. It consists of three areas that form the so-called inferior glenohumeral ligament complex: two bands (anterior and posterior) and the axillary pouch that spans between both bands (Fig. **1**). This disposition resembles a hammock for the humeral head to lie.

The humeral insertion is in the inferior and medial most part of the anatomical neck. In the glenoid rim, each band is inserted at different places; thus, if we consider the glenoid surface like a clock, the anterior band inserts in a right shoulder between 2:00 and 4:00 o´clock position and the posterior band between 7:00 and 9:00 o´clock position. The arrangement of the bands may vary: both can be parallel or can converge on the humerus having the same insertion point. The anterior band is usually thicker, while the posterior band is often wider [16].

During abduction, this ligament complex stabilizes the shoulder, but acts differently depending on the rotation of the arm: in internal rotation the posterior band tightens to support the head and the front side is idle and cordon-like. When the arm is positioned in external rotation, the anterior band tightens and unfolds supporting the head while the posterior band is folded. The posterior band also limits flexion, adduction and internal rotation [17, 18].

### 2.9 Labrum

The labrum is a fibrous ring that is intimately attached to the glenoid bone, the articular cartilage and the joint capsule. Histologically, it is composed of dense fibrous tissue. The long head of the biceps attaches to it in the superior part and the glenohumeral ligaments attach in the anterior part. In addition, it increases the functional depth of the glenoid, increasing the glenohumeral stability. Its absence decreases stability, but does not always cause clinical instability of the shoulder [19]. For example aging causes a clear deterioration of the labrum without a steady increase in clinical instability. Paradoxically, this does not mean that the older labrum does not have a stabilizing role, as it has been shown that it does keep this role regardless of age [20].

## 3 PHYSIOLOGY OF GLENOHUMERAL STABILITY

In this section, we describe the role each structure plays in shoulder stability and the properties of the joint that contribute to stability. We also describe the important contribution of proprioception in the joint control.

### 3.1 Ligamentous Stabilization

The shoulder ligamentous anatomy is clearly different from the knee ligaments, as there are no shoulder ligaments that maintain tension throughout the complete movement arch of the shoulder. Shoulder ligaments only act in the extreme positions of the range of motion (ROM), and remain lax if the shoulder is at rest or during non-extreme ROM [21]. During most of the ROM arch, there is no strain on the ligaments, these remain loose until a certain a point in which tension increases suddenly and quickly. The tense ligaments exert a compression force on the humeral head against the glenoid, further stabilizing the joint. Furthermore, there are personal variations in tension between individuals; subjects with increased laxity have greater degrees of rotation or translation but these do not need to be less stable [12, 22, 23]. Thus the ligaments provide a closed frame of glenohumeral joint displacement, inside this frame the muscles play the major stabilizing role.

### 3.2 Muscular Stabilization

There are different forces with different intensities and directions that act over he humeral head. These forces are delivered by the deltoid muscle, the tendon of the long head of the biceps and the muscles that make up the rotator cuff. Muscle orientation is key in defining force orientation. Under the direction of these muscles the force exerted on the shoulder is different. The sum of the vectors of these forces constitutes the net reaction force. If the net reaction force is directed into the glenoid, the joint remains stable. The greater the forces exerted toward the glenoid, the greater the stability of the joint. Regarding the forces exerted by the deltoid, the posterior and middle portions are more important in stabilizing the joint [24]. The posterosuperior rotator cuff (supraspinatus and infraspinatus) is essential as it produces a “ceiling” effect between the acromion and the humeral head that limits the upper translation even at rest [25]. When there is a deficit of any of these muscles, a misalignment of the net forces develops, increasing the chances of dislocation, as the net force may not be directed to the glenoid.

### 3.3 Bone Stabilization

The effective glenoid arch is the entire glenoid surface in which the humerus rests; this arch is formed by the glenoid cartilage, the underlying bone and the labrum. If this arch decreases for whatever reason, the chances of dislocation increase, as the possibility that the net reaction force is directed away from the glenoid increases. Glenoid anteversion can also increase the risk of instability because the net reaction force might be directed out of the glenoid.

Scapulo-thoracic movement is a key to obtain a broad degree of arm movement with the humerus aligned with the glenoid. If the glenoid does not follow the humerus during motion, the humerus can shift away from the glenoid center which increases the risk of instability. Whatever the cause, if the humeral head is not centered into the glenoid, the joint becomes less stable.

### 3.4 Physical Properties of the Joint that Provide Stability

These physical properties make the shoulder at rest to remain stable. These properties are adhesive effect, suction effect and negative pressure.

Adhesion between the glenoid and humeral head is due molecular attraction between the synovial liquid and the chondral surfaces of both the glenoid and the humerus. Degenerative or inflammatory diseases can affect this attraction effect by altering the molecular characteristics of the joint fluid [26].

The suction effect is due to the sealed environment that the labrum and the capsule ensure. This suction effect draws liquid and air out of the joint and resists traction.

There is a negative pressure produced by the synovial membrane that, through osmotic action, removes free joint fluid. This negative pressure causes a vacuum effect and gives stability to the joint. This effect is affected with arthroscopic techniques or arthrography but also by joint effusion [27].

### 3.5 Proprioception

roprioception is a summation of all the afferent stimuli coming out from the joint into the nervous system. As noted, after the appearance of the work of Turkel, special consideration was given to the static capsule-ligamentary structures as main stabilizers of the glenohumeral joint [1]. Since then, there has been an increased knowledge of the important role that the articular proprioception has in the shoulder, as it has an essential role in controling the correct activation of the active stabilizers. Studies on anatomical specimens have confirmed the presence of the corresponding afferent endings (Pacini and Ruffini corpuscles) and their distribution in the shoulder capsular-ligamentous complex [2]. These receptors monitor the movements and positions of the articular ends in space.

## 4 PATHOPHYSIOLOGY OF INSTABILITY

In addition to the lesion of the labrum and capsulo-ligamentary elements, other alterations may be present in other structures facilitating glenohumeral instability: glenoid or humeral bone injury or glenoid subscapularis tendon injury. Perhaps that is why many authors believe to have found the *“essential lesion”* of instability in the isolated involvement of some of these damaged structures.

**Fig. (2):**
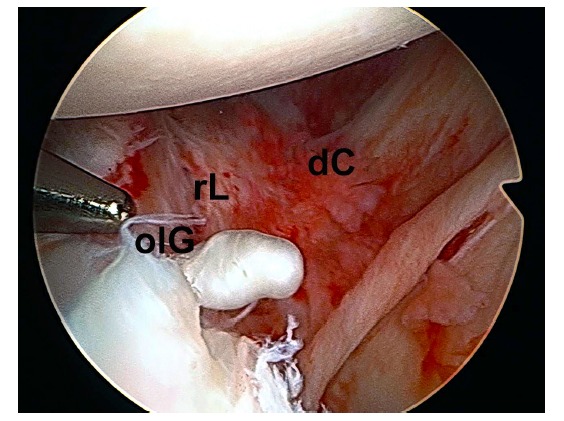
Arthroscopic view of a left shoulder in a patient with instability through a superior portal. Combined lesions of the anterior labrum, osseous lesion of the glenoid and capsular lesions (rL: retracted labrum; dC: distended capsule; olG: osseous lesion of the glenoid) can be aprecited.

However, it is now accepted that the cause of recurrent glenohumeral dislocation is a combination of anatomical lesions (inherent or acquired) of the so-called capsulo-ligamentous static factors. This combination of injuries causes, as we have noted, a further imbalance in the harmonic performance of different muscle elements that interact on the shoulder (an alteration of the so-called dynamic factors) [28]. Thus, the labral avulsion injury is often associated with excessive capsular laxity that promotes a pathological redundancy (Fig. **2**).

 Such redundancy is found in most cases of recurrent humeral dislocation and especially in more complex or multi-directional cases [28]. The excessive laxity that fosters pathological capsular redundancy can be even congenital in nature. There are however other causes of capsular laxity that are more common than congenital etiology: repeated micro-trauma, major trauma, multiple episodes or a combination of these [29].

 Furthermore, in some cases, these injuries of the anatomical structures may be associated with structural or morphological changes (collagen diseases, dysplasia...) and / or anatomical or congenital variants that also play a role in facilitating shoulder joint dislocation [30].

Let’s analyze the different elements involved in shoulder stability and how their variations or injury foster instability.

### 4.1 Anatomical Injuries. The “circle” Injury Concept

Stability results from the combination of four types of lesions [28]

a: Labral avulsion (Bankart lesion or its variants) with or without associated injury to the anterior band of the inferior glenohumeral ligament.
b: Plastic deformity of the capsule: irreversible elongation of the capsule or the inferior glenohumeral ligament.
c: Lesion by distension or elongation of the capsule in the rotator interval.
d: Humeral or glenoid bone dEfects.

**Fig. (3):**
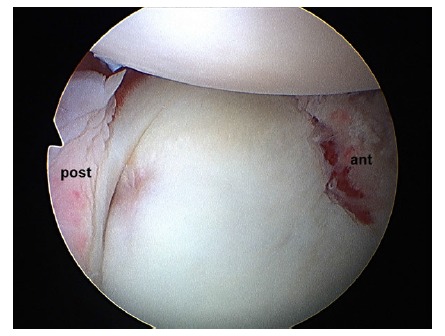
Arthroscopic view of a left shoulder in a patient with instability through a superior portal. Circle lesions can be appreciated with combined anterior (ant) and posterior (post) lesions.

For a dislocation to occur, lesions must be produced at least in two opposite parts of the joint (Fig. **3**). This phenomenon integrates the concept called “circle injury” described by Warren since 1984 [18, 31].

#### 4.2 Labral Avulsion and Inferior Glenohumeral Ligament

**Fig. (4):**
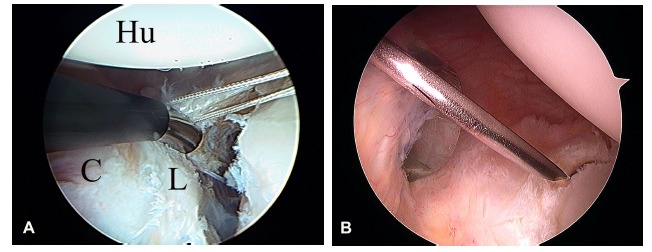
Arthroscopic view of a right shoulder in a patient with instability through a superior portal. **A)** Bankart lesion. Labral detachment (Hu: Humerus; L: Labrum; C: Capsule). **B)** Arthroscopic view of a left shoulder in a patient with instability through a superior portal. Posterior inverse Bankart lesion.

The labrum contributes to the stability of the joint by increasing the concavity and depth of the glenoid, dynamically acting as would a “wedge” breaking a wheel on a ramp [31]. Its injury during the episode of dislocation was originally described by Perthes and, separately, by Bankart (Fig. **4**) [7, 33]. It is different from the lesion described by Broca and Hartman, in this case the injury drags the periosteum of the anterior neck scapula alongside the labrum [33]. In either case, the detachment of the labrum leads to injury of the capsule and IGHL. Some anatomic variations of the arrangement of the labrum and its ligamentous attachments could encourage instability, as De Palma described [34].

#### 4.3 Capsular Plastic Deformity

The irreversible elongation of the capsule and inferior gleno-humeral ligament is a necessary condition of recurrent dislocation. The capsule behaves as a single structure and the development of an injury at one point is accompanied by an injury in the opposite capsule (“circle concept”) [18, 31]; it is a concept analogous to the “ring lesions” of the pelvis. Thus, shoulder instability cases often have more than one injury component. This association is considered as bi-directional type instability (anterior-inferior, posterior-inferior) or multi-directional type instability [35].

**Fig. (5):**
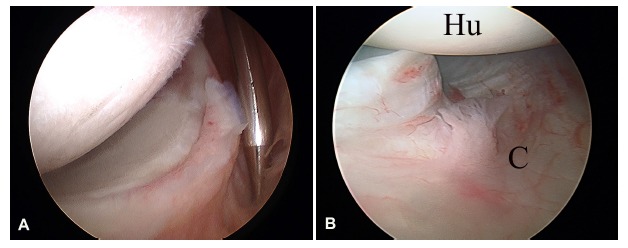
Arthroscopic view of capsular distension in the right shoulder of a patient with shoulder instability; **A)** View from supero-lateral portal view and, **B)** View from posterior portal view with a 30-degree scope (Hu: Humerus; **C**: Capsule).

Neer described the presence of a redundancy at the inferior part of the capsule in multi-directional instability studies [17]. However, the tension studies performed by Bigliani, Speer and Mc Mahon, among others, were the ones that showed that a plastic non-recoverable deformation of the capsule and the inferior glenohumeral ligament are paramount. The capsule can also have a congenital reduction of collagen fibers [36-40]. It is well known that these alterations lead to hiperlaxity,that is generally poly-articular. However, in most cases of recurrent dislocation only less pronounced forms of laxity are found; these can result in the presence of signs of joint hypermobility. The origin of the capsular laxity can be congenital or constitutional [30, 41, 42], but it can also be traumatic and acquired, perhaps the most frequent origin being a mix of inherent predisposition and a traumatic component (Fig. **5**) [35, 37-40, 42, 43]. We have previously noted that the capsule can also have anatomical variations in its insertion that can predispose to instability [30].

#### 4.4 Injury to the Rotator Interval

**Fig. (6):**
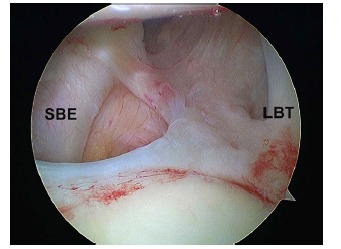
Arthroscopic view of a right shoulder in a patient with instability through a posterior portal. Attenuation and distension with synovial reaction is a frequent lesion of the rotator interval in multidirectional instability. (SBE: subscapularis tendon; LBT: longus biceps tendon).

Neer introduced the term “rotator interval” [36]. He defined a capsular area between the subscapularis and supraspinatus muscles extending in a triangle with a base at the coracoid. According to anatomical studies, this capsular structure is reinforced by two ligamentous structures: The superior glenohumeral ligament and the coracohumeral ligament [44]. Harryman showed that the rotator interval plays an important role in stabilizing the shoulder limiting inferior and posterior displacement [45]. There are two types of rotator interval injury: attenuation with synovial reaction or clear rupture (Fig. **6**) [46]. The rotator interval injury is necessary and concurrent to the development of an enlarged inferior capsular recess (“circle of injuries concept”) that is typical of recurrent dislocation with anterior-inferior directional component [18, 31].

#### 4.5 Glenohumeral Bone Defects

**Fig. (7):**
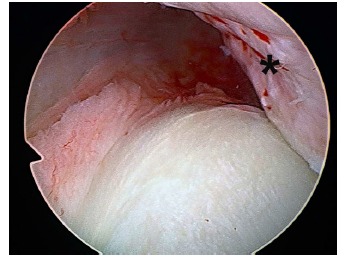
Arthroscopic view forn the posterior portal of a paitent´s left shoulder with an engaging “off-track” humeral lesion (asterisk).

Bone defects that occur in the glenoid and the humerus during episodes of dislocation contribute to recurrent instability. Instability due to glenoid defect happens in the middle range of motion, since in this range the stability is given by the glenoid concavity and the compression effect exerted by the shoulder musculature [47]. In contrast, humeral defect instability occurs at the ends of the range of movement, since it is then when the humeral defects can engage with the glenoid rim causing instability (Fig. **7**). Glenoid bone lesions occur already in the first episode of dislocation in 22% of cases [48], but in recurrent dislocation cases it can be seen in 90% of cases [49]. If surgery is performed during the acute phase usually bone fragments can be found [50], whereas if surgery is delayed for more than 6 months these fragments will often have been reabsorbed [51]. The humeral defect is in the posterior humeral head in cases of anterior instability, and it is known by the name of Hill-Sachs defect. This humeral defect occurs as the humerus engages against the glenoid during dislocation and reduction and is already present in up to 51% of patients after the first episode of dislocation. The bone loss has to be assessed carefully because, if is it not addressed properly during surgery, they can cause the surgery to fail. Patients without bone defects have a risk of recurrence around 4%, while for those who have bone loss the risk of recurrence can raise up to 67% [52].

A biomechanical study showed that a superior-inferior glenoid loss greater than 21% of the length implies a high risk of failure despite a good Bankart lesion repair [47]. Other clinicians consider a loss of 25% of the width of the glenoid as the critical size that requires additional attention and associated procedures to the Bankart surgical repair [53]. Therefore, a loss of superior-inferior length of 20% or a width loss of 25% or greater at the antero-inferior part of the glenoid would require performing an associated bone graft procedure [54].

## 4.6 Impaired Proprioception

There are two theories that attempt to explain this alteration: A) There would be an injury or neurological impairment of the elements constituting the proprioceptive reflex arc based on either the receptors themselves (mechanic-receptors) or the motor neurons that complete the reflex arc. B) There is primary injury and / or insufficient capsular-ligamentous stabilizing elements that hold the sensory afferent endings. This would cause a disturbance in the detection of pressure and elongation stress signals, and alteration of the signal would be transmitted by the sensory afferent channels While there is no contrasting works that prove the certainty of the first theory, the capsular injury (“the plastic deformity”) defined as “redundancy” has been confirmed by various authors, as already noted [37-40]. Consequently, a delay or lack of coordination of the corresponding muscle response occur [4, 5]. The results of various studies allow us to note that the capsular sensors are not altered or injured in instability [6, 55-58]. Nor are these altered by age or surgery. There is certainly an impairment of the proprioceptive function in patients with unstable shoulders showed by different authors by a delay in the detection of passive movements, especially of external rotation but it is not related to changes in the sensors [6, 55-58].

In summary, proprioceptive dysfunction would be produced by a delayed signal production due to structural alterations at the capsule [4, 5]. In the unstable shoulder an injury and / or failure of the passive capsulo-ligamentary stabilizing elements would have occurred. At this level is where the sensory afferent endings are located. Due to this injury a disturbance in the detection of pressure signals, stress and elongation is transmitted altered through the afferent sensory channels; this disturbance takes the shape of delayed and / or uncoordinated muscle response, causing the loss of joint congruity. This would be the patho-mechanical chain disturbance that alters the proprioception mechanism and causes instability or recurrent dislocation chain. The picture would worsen with increased capsulo-labral damage and widening of the capsular recess. This hypothesis (delay in production of the signal without significant injury of the proprioception sensors) has been conclusively corroborated by the work of Zuckerman that confirms the recovery of the impairment after surgery [59].

## 5 THERAPEUTIC IMPLICATIONS

### 5.1 Of the Capsular Injuries

An arthroscopic or open surgery failure is mostly related to incorrect evaluation and / or inssuficient correction of instability in the inferior part of the joint. These mistakes are due to the difficulty of assess the importance of capsular injury and the difficulty of some surgical techniques that can deal with that capsular injury. To consider that it is sufficient to repair the Bankart lesion can be a cause of failure. Several studies indicate that an isolated avulsion of the labrum (Bankart lesion or its variants) produces only small shifts anteriorly [21, 38, 60]. Therefore, the latest surgical techniques seek actively a joint volume reduction by capsular plication or anterior capsular shift [61]. Patients with larger labral lesions should be expected to have more significant capsular injury and larger capsular recesses that have an important impact in joint proprioception. Therefore a reduction of capsular volume should be always attempted according to the latest arthroscopic surgical techniques. To achieve adequate surgical outcome and avoid technical errors, it is necessary to control that component of redundant capsular volume. However, it is difficult to quantify the degree of injury of the capsular-ligamentous structures that cause this inferior instability, making it difficult to define the adequate capsular reduction [35, 37, 42, 62].

There are also other factors that increase the complexity of the problem: 1) in some patients the normal humeral head may move between 1 and 2 cm in any direction under normal circumstances [61], in these cases even small capsular elongation injuries may cause the emergence of symptoms [62]. 2) Lesions of the rotator cuff tendons may also allow for a significant increase in the normal displacement of the humeral head [12, 63, 64].

One alternative is to directly assess the displacement of the humeral head (DH) during arthroscopy. This allows the examiner to assess displacement in different directions and can help in defining the importance and the degree of involvement of the capsular component in each case. Repeated measurements after the different surgical steps can allow us to establish if the objectives in capsular volume reduction have been obtained. The objective should be to replicate the displacement found in shoulders without significant instability problems [64].

### 5.2 Of Bone Lesions

It is accepted that antero-inferior defects larger than 25% of the width of the glenoid must be operated by bone grafting techniques or Latarget-type coracoid transfers [54-66]. Bone grafts are used to rebuild the glenoid articular curvature and depth. Coracoid transfers seek a reconstruction of the anatomical concavity and specifically an anterior mechanical stabilization effect [67]. As for humeral Hill-Sachs lesions, lesions larger than 25% of the perimeter of the humeral head might require allograft reconstruction [68]. More recently an infraspinatus tenodesis in the defect (the “*remplissage*” technique) has been proposed as an alternative in these cases [69].

In order to assess the need for more aggressive procedures to deal with bone defects it is necessary to accurately assess the increase in risk that their presence carries. To completely assess this risk we must evaluate not only the defect size and orientation in each bone, but the relationship between humeral and glenoid defects during movement of abduction and external rotation of the arm. It is necessary to assess both bone defects as a whole [52]. Thus, when the humeral defect is parallel to the glenoid rim with the arm in position of abduction and external rotation the risk of engaging is higher, the so called “Engaging Hill-Sachs lesion”, which carries and extremely high risk of recurrence [52].

In recent publications, Burkhart himself has acknowledged that to accurately assess risk it is important to measure the relation between the glenoid rim and the humeral head and, most importantly, the effect that the defects have on it. This is the so called “glenoid track” [70, 71]. It requires to evaluate the part of the humeral head that contacts the glenoid during external rotation and abduction; a zone that extends from the infero-medial part of the posterior humerus to the supero-lateral part [60].

The key factor is then the location of the medial margin of Hill-Sachs regarding the glenoid rim. If the Hill-Sachs defect is fully covered by the glenoid throughout the range of motion, it is considered “on-track” and there is no risk of dislocation. However, when the Hill-Sachs lesion is found medial to the glenoid projection, regardless of the depth and size, it is considered “off-track” and has a high likelihood of engagement.

**Fig. (8):**
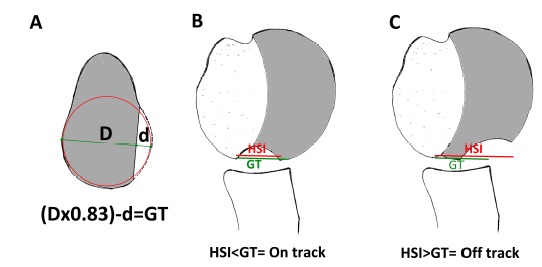
**A:** The “Hill-Sachs Interval” (HSI) is the distance from the footprint of the sleeve to the medial margin of the lesion of Hill-Sachs. In a normal shoulder 83% of the diameter of the glenoid (**D**) corresponds to the distance between the cuff footprint and the medial margin of the glenoid, this distance is the “glenoid track”. If there is a glenoid defect (**d**), the defect must be subtracted form 83% of the glenoid width to get the true Glenoid track (GT=(D*.83)-d). **B**: An engaging lesion combination or injury “off-track”. The HSI is greater than the GT, the medial margin of Hill-Sachs is located medial to the projection area of ​​the glenoid rim glenoid producing engagement. C: Non engaging lesion combination or injury “on-track”. With no glenoid defect, the HSI will be smaller than the GT, the Hill-Sachs will be covered by the glenoid at all times and there is no risk of engaging.

The exact location of the Hill-Sachs lesion relative to the glenoid can be measured in a CT scan by making a comparison with the contralateral shoulder; it can also be assessed arthroscopically. The distance from the edge of the footprint area of the rotator cuff to the medial margin of Hill-Sachs has to be calculated, that is the width of the Hill-Sachs plus the distance between it and the footprint of the cuff. This distance is called the Hill-Sachs interval (HIS) (Fig. **8A**).

On the other hand we must calculate variations in the glenoid track (GT). 83% of the glenoid width corresponds to the distance between the cuff footprint and the medial margin of the glenoid projection If there is a glenoid defect, this defect must be subtracted from the 83% of the glenoid width for the true glenoid track. Knowing this glenoid track we can calculate in the humerus where the medial margin of the glenoid area projection should be located (8a) [71].

Knowing the HSI and the GT we can calculate the real risk of fouling. When the HSI is greater than the GT, the medial margin of the Hill-Sachs will be medial to the GT, which will produce engaging; this is considered injury outside the area or “off-track” (Fig. **8B**).

When there is no glenoid defect or this is small, the HSI will be smaller than the GT, so the Hill-Sachs will be covered by the glenoid at all times, that is, will be “on-track” and there will be a reduced risk of engagement (Fig. **8C**). In this way we can correlate the two bone lesions intra-operatively or in imaging to select the correct surgery. In cases where an “off-track” glenoid tracking is identified additional procedures should be associated to the Bankart repair, for example a “*remplissage*” could be performed even with a humeral defect > 25% [71]. Similarly bone grafting has the effect of increasing the area of glenoid track and thus tuning “off-track” shoulders into “on-track” shoulders [52, 71].

## CONCLUSION

The correct treatment of recurrent dislocation is linked to an accurate knowledge of the pathogenesis and the development of increasingly specific exploration tests [72, 73]. It is important to bring surgical treatment closer to the earlier stages in the evolution of the disease, detecting patients that at risk of recurrence and that won’t be successfully dealt with conservative treatment [74, 75].

## LIST OF ABBREVIATIONS

(GHL): = Gleno Humeral Ligaments
(GT): = Glenoid Track
(HIS): = Hill-Sachs Interval
(DH): = Humeral Head
(IGHL): = Inferior Glenohumeral Ligament
(ROM): = Range of Motion
